# Advances in Mammalian Metallomics: New Insights into Metal Dynamics and Biological Significance

**DOI:** 10.3390/ijms26199729

**Published:** 2025-10-06

**Authors:** Xin Tian, Yifan Teng, Yuhang Deng, Qian Zhang, Caihong Hu, Jie Feng

**Affiliations:** 1 Key Laboratory of Animal Nutrition and Feed of Zhejiang Province, College of Animal Sciences, Zhejiang University, Hangzhou 310027, China; 2 Key Laboratory of Nutrition and Breeding for High-Quality Animal Products of Zhejiang Province, College of Animal Sciences, Zhejiang University, Hangzhou 310027, China

**Keywords:** metallomics, mammals, metal dynamics, biological functions, absorption and metabolism, metal detection technologies

## Abstract

Mammalian metallomics, an advanced interdisciplinary field, explores the dynamic roles of metal elements within biological systems and their significance to life processes. While prior reviews have broadly covered metallomics across different systems, this review narrows the focus to mammals, offering new insights into the physiological roles of metal elements, their complex absorption and transport mechanisms, and their intricate associations with diseases. We summarize the characteristics and applications of common metal detection technologies and elaborate on the dynamic landscape of the mammalian metallomics across different tissues and life stages. Furthermore, we elaborate on the physiological functions of the metals from three perspectives, metal-binding proteins, metal ions, and gut microorganisms, and highlight the potential of metallomics in clinical translation, including its diagnostic and therapeutic implications, alongside future directions centered on multi-omics integration. Overall, this review introduces several common metallomics technologies and synthesizes the findings of mammalian metallomics research from multiple perspectives, offering new insights for future related studies.

## 1. Introduction

Metallomics is a significant interdisciplinary field that explores the intricate roles of metal elements in biological systems, focusing on metal homeostasis, advanced analytical methodologies, and the biological significance of metals [1,2]. Metal detection has been extensively applied in the determination of metal element content in non-living materials such as soil for a considerable period, primarily due to the assessment of metal element toxicity and environmental pollution [3,4]. This approach is crucial for detecting the contents of metal elements within soil and further elucidating the translocation and conduction of these elements within plant organisms [5,6]. However, the advancement in nutritional studies and metal analytical techniques has helped researchers to delve deeper into the distribution and function of metal elements within animals, especially mammals.

Metal elements are vital components of biomolecules and participate in numerous physiological processes [7]. Elements such as iron [8], zinc [9,10], and copper [11] are essential for various physiological functions, including enzyme activity, oxygen transport, and cellular growth. Additionally, disruptions in the homeostasis of metal elements are intimately linked to a multitude of diseases, such as neurodegenerative conditions like Alzheimer’s disease [12] and Parkinson’s disease [13], which demonstrated aberrations in metal metabolism. Thus, in-depth studies on the dynamics and biological functions of metals are crucial for comprehending life processes and disease mechanisms in mammals. There are a multitude of metal elements in mammals, including essential trace metal elements with a low content of total mass, such as iron, zinc, copper, and manganese, which play an indispensable role in physiological functions [7,8,9,10]. Simultaneously, macroelements such as calcium, magnesium, sodium, and potassium are key factors for maintaining the basic life activities of animals [14]. They play a foundational role in maintaining the fluid balance in animal bodies, constructing skeletal structures, and transmitting neural signals [15].

The relationship between metal imbalance and metal ion transport disorders with diseases has increasingly garnered attention. Studies indicate that aberrant concentrations of metal elements may lead to functional impairments in cells, thereby triggering a cascade of diseases [16,17,18]. Additionally, impaired metal transport can lead to systemic ion dyshomeostasis, resulting in various complications [19]. For instance, dysregulation of copper and zinc metabolism is directly associated with the onset of Alzheimer’s disease [20]. And both excess and deficiency of iron and zinc can lead to various health issues, including anemia, compromised immune function, cardiovascular diseases, impaired growth and development, skin damage, and poor development of sexual organs [21]. Meanwhile, there are variations in the contents of metal elements across multiple organs during various stages of the mammalian life cycle [22], and researchers have shown that these dynamic changes are closely related to life processes such as lactation and aging [23]. Understanding the roles of metals in mammals and their physiological impacts is vital for developing treatments to restore metal balance and prevent diseases, as well as for implementing precision nutrition strategies.

Therefore, this review outlines the progress in mammalian metallomics from five perspectives: the advancements in metallomics technologies, the physiological functions of metals, the mechanisms of metal transport and metabolism, the dynamic landscape of metals, and the future directions of metallomics research.

## 2. The Advancements in Metallomics Technologies

**Figure 1:**
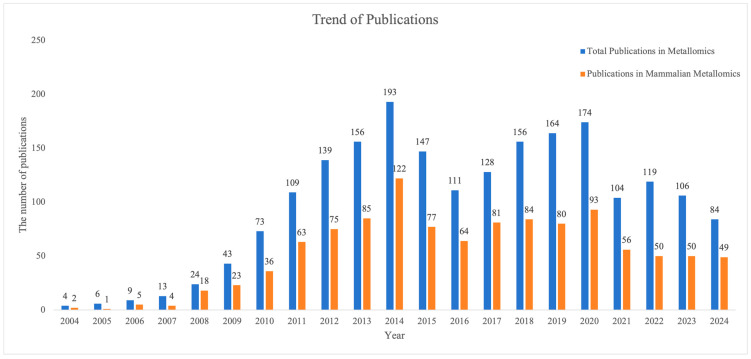
Publication trends of metallomics and mammalian metallomics. The publication data was retrieved from the Web of Science Core Collection using the topic search query TS = ("Metallomics") combined with representative mammal terms (e.g., Human, Mice, and Rat), published through 31 December 2024.

Metal detection techniques have achieved remarkable progress in recent years, broadening the application range of metallomics. While the total number of metallomics publications has stabilized, Figure 1 shows that mammalian metallomics has consistently accounted for a large share of the literature over the past fifteen years. This enduring prominence underscores its central role in biomedicine and life sciences research. Increasing numbers of metallomics technologies have emerged, including the detection of metal elements and metal isotope determination in samples via mass spectrometry and spectroscopy [24,25] and the structural determination of metal complexes using spectroscopy, crystallography, and cryo-electron microscopy [26,27]. Mass spectrometry techniques and emerging imaging technologies are especially widely used for detecting metal distribution in tissues and understanding the mechanisms of metal elements in biological systems [24,25]. Coverage of metallomics techniques applied in mammalian research will be systematically reviewed in the following subsections, with key findings conclusively summarized in Table 1.

### 2.1. Application of Spectroscopy in Metallomics

Spectroscopy analyzes sample composition and concentration via light–matter interaction [28,29,30], with three primary metallomics applications:

Atomic Absorption Spectrophotometry (AAS): AAS is commonly used for metal analysis [30], including FAAS (detection limit: high ppb-ppm) and GFAAS (sub-ppb; higher sensitivity/cost), and is applied to animal samples like milk [24], serum [31], feces [32], and tissues [33] and in lactation studies [34,35] but is limited to single-element measurement per experiment.

Inductively Coupled Plasma Optical Emission Spectrometry (ICP-OES): ICP-OES utilizes plasma ionization for simultaneous multi-element detection at 1–10 ppb sensitivity [36,37,38], offering high resolution and clinical utility in nutrition/medicine [39], such as adrenal adenoma tissue analysis showing elevated Mn, Cu, and Zn [38].

X-Ray Fluorescence (XRF): XRF enables non-destructive, multi-element analysis from percentage to sub-ppm concentrations through X-ray-induced fluorescence [40,41], ideal for reusable samples in plant science [42], archaeology [43], and mammalian studies including milk and liver specimens [44,45].

### 2.2. Application of Mass Spectrometry in Metallomics

Mass spectrometry identifies compounds by measuring ion mass-to-charge ratios [43] where molecules are ionized and separated for detection [46]. Technological advances have deepened insights into the roles of metals in biological processes and biomedicine. Inductively Coupled Plasma Mass Spectrometry (ICP-MS) serves as a cornerstone of metal detection [45], offering unparalleled sensitivity (ppt-level detection limit) and wide linear dynamic range for precise metal/isotopic quantification in complex samples [47,48] yet is constrained by complex pretreatment (e.g., nitric acid digestion for liver/feces) and high costs [45,49]. Its vital applications span basic medicine and pharmacology, enabling blood metallomics analysis for ASD biomarker discovery [50] and revealing metal-based anticancer drug distribution in tumors to advance pharmaceutical development [51].

### 2.3. Application of Imaging Techniques in Metallomics

Emerging imaging techniques enable spatial mapping of metal distributions in biological tissues [52,53], revolutionizing metallomics through integrated methods like IMC-LA-ICP-TOFMS that reveal cellular heterogeneity via metal-tagged antibodies. This approach maps sodium/iron homeostasis in tumors, linking iron-enriched regions to vascular distribution and proliferation for targeted drug delivery [53]. Predominantly applied LA-ICP-MS [25,54] employs laser ablation sampling, plasma ionization [55], and mass-filter detection to generate 2D/3D elemental distribution maps [25,47,56,57,58], visualizing concentration gradients in solid samples across geology, biology, and medicine. Critical applications include mapping lead distribution (uniform in liver vs. heterogeneous in brain/kidneys) [59] and uranium/thorium organ-specific accumulation (renal uranium vs. hepatic thorium) [25], advancing toxicokinetic studies.

### 2.4. Challenges in the Standardization of Metal Detection

Despite the significant advancements in metallomics research, the field currently faces challenges related to the lack of standardized methodologies. For instance, in ICP-MS analysis, variations in sample preparation protocols, such as differences in digestion time and temperature or whether samples are lyophilized prior to digestion, can significantly impact the results. These discrepancies can affect the comparability and reproducibility of findings across studies. Similarly, imaging techniques used in metallomics lack standardized parameters, making it difficult to integrate and compare image data across studies. Thus, addressing these limitations through the development of standardized protocols is crucial for the advancement of mammalian metallomics research.

## 3. Physiological Functions of Metal Elements in Mammals

**Figure 2:**
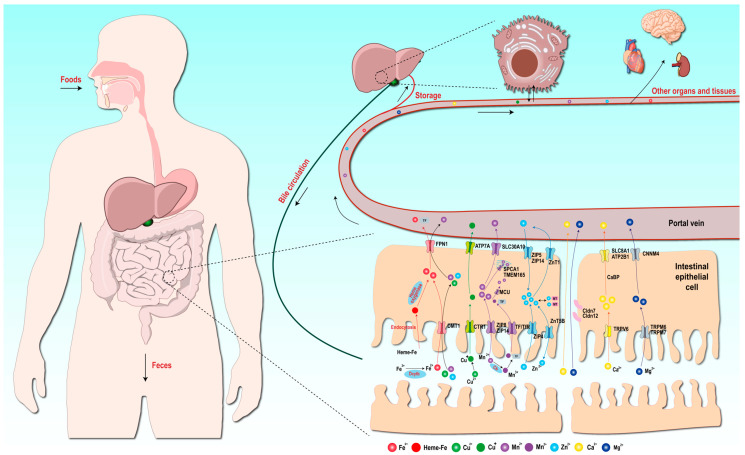
Systemic transport and metabolic homeostasis of essential trace elements in organisms. Synthesizing multi-element transport and metabolic pathways within mammals.

Metals play crucial physiological roles in mammals, acting as essential cofactors for many enzymes and participating in various cellular metabolic processes [60]. They are also vital for the survival, growth, and development of organisms [9,61]. Metals are integral to the formation of biological structures such as skeletal systems that provide support for vital life activities. Moreover, approximately 30% of the total proteins in organisms are metal-binding proteins (MBPs), which are intimately connected with the life-sustaining activities of the organism [27,62]. The roles of macro minerals such as K, Ca, Na, and Mg in the body have been extensively reviewed [63,64,65,66], and this section will focus on the trace elements iron (Fe), copper (Cu), zinc (Zn), and manganese (Mn) given that they represent some of the most extensively studies in mammalian metallomics, with well-characterized mechanisms of absorption, transport, and physiological function, alongside systematically investigated associations with various diseases. However, it is acknowledged that elements such as cobalt (Co), molybdenum (Mo), and nickel (Ni) also play critical physiological roles [67]. Cobalt (Co) is a central component of vitamin B12 [68], molybdenum (Mo) participates in sulfide metabolism and detoxification [69], and nickel (Ni) serves as an essential cofactor for urease in certain microorganisms like Helicobacter pylori [70,71]. The exclusion of these elements from a detailed analysis herein is not an indication of lesser importance but rather a reflection of the current focus and scope, with the expectation that future research will expand into a more comprehensive landscape. Figure 2 provides a concise synthesis of how essential trace elements (Fe, Cu, Mn, and Zn) execute physiological functions via these mechanistic categories.

### 3.1. Biological Functions of Metal Elements

Trace elements, which constitute less than 0.01% of an organism’s body weight, encompass essential transition metals, including iron (Fe), copper (Cu), zinc (Zn), and manganese (Mn) [61,72]. These trace metal elements are indispensable for the survival and health of organisms and participate in various life activities of the organism in multiple forms. Trace elements, as essential cofactors for many enzymes and proteins, are involved in catalyzing a variety of chemical reactions in living organisms and assist in life activities such as the digestion of nutrients, the transportation of nutrients, and the synthesis of genetic materials [73,74,75]. Metal elements participate in the life activities of mammals and primarily execute biological functions through three forms: MBPs and enzymes, metal ions, and modulation of gut microorganisms.

(1) Metal elements perform biological functions through MBPs and enzymes

Metal ions are the functional core of a vast array of enzymes and metal-binding proteins (MBPs) and frequently act as the key reactive centers. The specific role a metal plays is intricately linked to its distinctive physicochemical profile, including redox activity, coordination geometry, and Lewis acidity, enabling it to perform precise catalytic or structural functions [76,77,78].

Iron, as one of the most essential trace elements in animals, is involved in the composition of various MBPs, such as hemoglobin, ferroprotein (FPN), transferrin receptor (TFR), ferritin, and mitochondrial proteins [79]. These proteins play crucial roles in many key physiological processes within organisms, ranging from oxygen transport to energy metabolism, and from iron storage to utilization [80,81]. Iron serves as the active center of many enzymes, such as peroxidase [82,83] and tryptophan dioxygenase [84], participating in a series of metabolic activities, including ribosomal biosynthesis, protein translation, lipid metabolism, and mitochondrial oxidative phosphorylation [85]. Iron is also an essential cofactor for key enzymes in DNA metabolism, including various DNA repair enzymes (nucleases, glycosylases, demethylases) and ribonucleotide reductase [86,87]. These enzymes are crucial for maintaining the stability and integrity of the genome. The ability of iron to adopt multiple oxidation states underlies its crucial role in biological redox processes. In cytochromes, iron cycles between Fe^2+^ and Fe^3+^ to mediate electron transport. In monooxygenases like cytochrome P450, the heme-iron center activates molecular oxygen (O_2_) for the oxidative transformation of substrates, including reactions essential to drug metabolism and hormone synthesis [88,89]. This redox versatility makes iron uniquely suited for these functions.

Similar to iron, copper is also a component of numerous MBPs and enzymes such as ceruloplasmin (CP), cytochrome c oxidase, and Cu-SOD [90,91]. These proteins and enzymes are involved in essential physiological processes, including electron transport, oxygen metabolism, and antioxidant defense mechanisms [92]. For instance, cytochrome c oxidase is a key enzyme in the mitochondrial electron transport chain, facilitating cellular respiration and energy production [92,93]. Copper serves as a cofactor for a variety of enzymes, enabling them to catalyze important biochemical reactions [94]. Examples of these enzymes include lysyl oxidase, which is involved in the cross-linking of collagen and elastin in connective tissues [94,95], and dopamine β-hydroxylase, which is crucial for the synthesis of norepinephrine and relies on copper for their catalytic activity [96]. Copper-dependent enzymes are also involved in iron metabolism, as seen in the function of ceruloplasmin, which oxidizes ferrous iron to ferric iron, facilitating its transport in the bloodstream [97]. Copper enzymes play a pivotal role in molecular oxygen metabolism and free radical regulation. For instance, superoxide dismutase 1 (SOD1) employs both copper and zinc ions, with the copper ions serving as the catalytic core. The Cu center undergoes reversible redox cycling between Cu^2+^ and Cu^+^ to catalyze the disproportionation of superoxide radicals (O_2_^−^) into molecular oxygen and hydrogen peroxide, constituting a critical component of the cellular antioxidant defense system [98,99]. And, in cytochrome c oxidase, the terminal enzyme complex of the mitochondrial respiratory chain, copper ions (along with heme iron) facilitate the controlled reduction of oxygen. Specifically, binuclear copper center (Cu_a_) and hemeα_3_ work in concert at the enzymes’ active sites to catalyze the four-electron reduction of O_2_ to H_2_O. This process is coupled with proton pumping across the mitochondrial membrane, thereby contributing to the electrochemical gradient essential for ATP synthesis. The presence of a multi-metal active site allows a stepwise, energetically favorable transfer of electrons, thereby minimizing the generation of toxic reactive oxygen species during oxygen activation [11,99].

Zinc is also an indispensable trace element. Zinc-binding proteins play a crucial role in regulating intracellular zinc ion concentrations. When the concentration of zinc ions within cells is high, the synthesis of zinc-binding proteins such as metallothionein increases to bind excess zinc ions, thereby protecting cells from the effects of zinc toxicity [100]. Furthermore, zinc is a cofactor for over 300 enzymes like carbonic anhydrase, Zn-SOD, and Gamma-Glutamyl transferase (GGT) [26,101]. Zinc plays a pivotal biological role in protein and carbohydrate metabolism, DNA and RNA synthesis, and immune regulation. [102,103,104]. And in contrast to iron, zinc is redox-inert but a potent Lewis acid. This property is exploited in hydrolytic enzymes like carbonic anhydrase and zinc-binding enzymes. In carbonic anhydrases, Zn^2+^ coordinates a water molecule, lowering its pK_a_ and generating a nucleophilic hydroxide ion (-OH) that attacks CO_2_. This mechanism demonstrates how zinc activates water for efficient catalysis without itself undergoing oxidation or reduction [105]. Additionally, zinc plays a crucial role, as seen in zinc finger proteins, where it coordinates amino acid residues to fold into stable domains that are essential for DNA recognition.

Manganese is a component of many important enzymes and proteins, such as arginase, pyruvate carboxylase, and RNA polymerase [15,93]. By mediating the production of these enzymes and proteins, manganese plays a crucial role in many physiological activities such as promoting bone development, facilitating carbohydrate and lipid metabolism, exhibiting antioxidant properties, and functioning in the immune and reproductive systems [106]. For instance, researchers found that high-fructose diet in mice lowers liver manganese and blood ammonia clearance, with fructose affecting these via the ChREBP/SLC30A10 pathway [107]. And analogous to the Cu-Zn cofactor in SOD1, manganese serves as the essential redox-active metal in Mn-superoxide dismutase (Mn-SOD), which similarly catalyzes the disproportionation of superoxide radicals (O_2_^−^) into oxygen and hydrogen peroxide, thereby providing a critical antioxidant defense within the mitochondrial matrix [108,109]. Although manganese has not been as extensively studied as iron, copper, or zinc, it still plays a significant role in maintaining health.

(2) Metal elements perform biological functions through metal ions

Metal ions represent a crucial form through which the metal elements in mammals carry out various biological functions. They exert their biological roles via a kinetically labile, readily exchangeable pool which is often operationally referred to as free ions rather than as truly free aquated species. For iron in particular, the consensus is that bare Fe^2+^ ions are essentially undetectable in the cytosol because every potential ligand (peptides, nucleotides, metabolites) will out-compete water for coordination [76,77,78]. Consequently, metal ion functions described in this section refer to reactions arising from the labile pool, not from thermodynamically free ions.

Iron ions can promote the proliferation and differentiation of immune cells [110], and iron-dependent histone H3K9 demethylation plays a significant role in B cell proliferation and humoral immune function [111,112]. Copper ions can modulate the activity of certain enzymes and are involved in the detoxification of reactive oxygen species through enzymes like SOD [113,114]. The redox properties of copper ions are essential for their role in electron transfer reactions and the maintenance of cellular redox homeostasis. Zinc ions are not only involved in cell proliferation and differentiation [115] but also play a role in antioxidant defense by aiding in the clearance of reactive oxygen species (ROS) from the body [101,116]. Additionally, zinc ions act as a second messenger within cells, participating in various signaling cascades [117]. Cellular activation is often accompanied by the consecutive release of zinc from different intracellular compartments. Manganese ions play a crucial role in the regulation of intracellular homeostasis and are critical for the cGAS-STING pathway. Mn ions activate the cGAS-STING pathway by enhancing cGAS DNA sensitivity and cGAMP synthesis following viral-infection-induced release from cellular organelles [74].

Thus, metal ions particularly trace element metal ions execute a variety of biological functions, including signal transduction, cellular metabolism, and catalytic reactions.

(3) Metal elements perform biological functions through gut microorganisms

Metal elements can maintain the metal homeostasis of mammals under different conditions by mediating the composition of the gut microbial community and influencing the production of its metabolites. Diet is a significant source of metal elements for mammals, but a considerable portion of these metal elements is not absorbed by the host and remains in the intestinal lumen for microbial utilization [102,118]. The homeostasis of the gut microorganisms contributes to the protection of the host’s intestinal barrier function and enhances immune function [18,119]. However, the interactions between the metal elements and the gut microorganisms vary greatly, and it remains unclear whether there are interactions between the gut microorganisms and certain metals [120]. This section will elaborate on the established relationship which has been reported between metals and gut microorganisms.

Only 5–20% of dietary iron is absorbed by the duodenum, implying that approximately 80% of ingested iron remains in the intestinal lumen (primarily in the colon) for utilization by microorganisms [121]. The gut microorganisms influence host iron absorption, while the host’s iron intake, iron deficiency, and iron excess affect bacterial biodiversity, taxonomy, and function, leading to changes in bacterial virulence [120,122]. For instance, iron deficiency may result in an increase in lactobacilli [123]. Additionally, gut microbes can affect iron absorption and metabolism by altering the intestinal environment (such as pH and redox state). Microbial metabolites such as short-chain fatty acids can also enhance the iron absorption capacity of intestinal epithelial cells [119,124]. Iron mediates the gut microbiota to maintain host iron homeostasis. The balance of iron is of vital importance for maintaining intestinal health and preventing related diseases.

The precise content and absorption rate of copper in the diet remain undefined. However, copper absorption is analogous to that of iron, predominantly in the duodenum [125]. Copper not absorbed by the host accumulates in the intestinal lumen until it is excreted with feces. The antimicrobial properties of copper imply that its intake and bioavailability directly influence the composition and function of the gut microbiota [126]. It was observed that weanling pigs fed with copper-supplemented diets exhibited an increased abundance of microbial communities associated with transcription mechanisms and tuberculosis [127]. Additionally, copper deficiency results in a reduced abundance of copper-tolerant bacteria, such as *Salmonella enterica*, *Cryptococcus* spp., and *Escherichia coli*, which are related to fatty acid degradation and carbohydrate metabolism [128,129].

The role of zinc in the gut microbiota is similar to that of iron and copper, although its absorption mechanisms and biological functions are different. These essential metal elements ingested by animals mainly originate from the diet. The absorption rate of dietary zinc in the human body varies among individuals and is usually between 25% and 35% [130]. Zinc is closely related to the metabolic activities of the gut microbiota. Physiological and nutritional doses of zinc can improve the integrity of the intestinal wall and reduce the entry of bacteria and gut microbiota metabolites into the systemic circulation [9]. Zinc supplementation has been found to increase the abundance of Gram-negative aerobic bacterial groups in the gut, especially the concentration of short-chain fatty acids (SCFAs), as well as the overall species richness and diversity [9,131]. Meanwhile, excessive zinc supplementation may increase the toxin levels of certain bacteria such as *Clostridium difficile* (*C. difficile*) and exacerbate infections [132,133]. On the other hand, zinc deficiency can lead to a decrease in the abundances of genera such as Blautia, Bifidobacterium, and Verrucomicrobia, which are mostly associated with the production of short-chain fatty acids and mucin [134,135].

As a trace element, manganese has not been as widely studied as iron, copper and zinc. Manganese, as a crucial cofactor for bacterial survival, can protect bacteria from reactive oxygen species, radiation, and acidic environments [136,137]. Manganese exposure can affect the abundances of gut microbiota, including bacterial phyla such as Shigella, Ruminococcus, Ruminococcaceae, and Streptococcaceae [138,139]. Meanwhile, high doses of manganese can disrupt the homeostasis of the gut microbiota and then lead to enterocyte cytotoxicity and also affect the integrity of the intestinal wall by destroying tight junctions [138]. The role of manganese in the gut microbiota is complex as it can not only influence the composition and function of the microbiota but also may affect the host’s health through the gut microbiota.

### 3.2. The Association Between Metal Imbalance and Diseases

The metal balance within living organisms is of paramount significance for the preservation of health. In recent years, a growing body of research has indicated that metal imbalance is intimately correlated with the onset of diverse diseases. Several factors, including malnutrition, diseases, and medications, can affect metal metabolism and absorption. The disturbance may upset the body’s homeostasis and lead to various pathological conditions [77]. This subsection will outline the associations between multiple metal imbalances and diseases, with information summarized in Table 2.

Neurodegenerative disorders can lead to the malfunction and demise of nerve cells. Common neurodegenerative ailments, including Alzheimer’s disease (AD), Parkinson’s disease (PD), Huntington’s disease (HD), and multiple sclerosis (MS), have been found to be associated with aberrant metabolism of metal elements like iron, copper, and zinc. Excessive levels of Cu^2+^ in the body can bind to amyloid-β (Aβ) peptide, facilitating the aggregation of Aβ and consequently resulting in AD [140]. Abnormally elevated concentrations of copper ions have been detected in the striatum of HD patients and in HD mouse models [11]. The augmentation of Fe deposition in the deep gray matter of the brain is regarded as an overall indicator of neurodegeneration in MS. Fe exhibits focal accumulation in the substantia nigra of PD patients, whereas in AD patients, diffuse accumulation of Fe occurs in regions such as the cortex and hippocampus [141]. Zinc deficiency can precipitate an imbalance between the functions of helper T cell (Th 1 and Th2), as well as between T regulatory (Treg) and pro-inflammatory T cells, and the failure of Th17 downregulation, which represents the primary mechanism underlying the development of MS [142]. Prolonged exposure to excessive Mn can lead to its accumulation in the brain and is correlated with dysfunction of the basal ganglia system, giving rise to severe neurological disorders akin to PD. The dysregulation of Mn metabolism in AD patients and the impairment of the Mn-SOD clearance system are linked to the formation of senile plaques [141].

Ferroptosis, a form of cell death induced by oxidative stress damage due to iron overload, has been demonstrated by numerous studies to be involved in the occurrence and progression of multiple diseases. In addition to the aforementioned neurodegenerative diseases, cardiovascular disorders such as heart failure and coronary heart disease, as well as chronic metabolic conditions like diabetes and hyperlipidemia, have been verified to be closely related to ferroptosis [143]. In comparison to non-malignant cells, the proliferation of cancer cells is highly reliant on iron. Inducing targeted ferroptosis to suppress the growth of tumor cells constitutes an efficacious approach for cancer treatment [144]. Copper serves as a crucial factor in cell signaling and participates in the carcinogenic process by promoting cell proliferation, angiogenesis, and metastasis. Chronic exposure to elevated levels of Cu in drinking water can stimulate cancer cell proliferation in a pancreatic neuroendocrine tumor model [145]. Copper can also augment the metastatic potential of cancer cells by activating metabolic enzymes and proliferative enzymes [146]. Zinc fulfills catalytic, structural, and regulatory functions within the body, rendering it indispensable for normal physiological operation. Many cancer patients, particularly those with lung cancer, breast cancer, head cancer, or neck cancer, exhibit reduced zinc levels in their blood [147]. Sodium and potassium are essential metal elements for maintaining cell homeostasis. Abnormal concentrations of sodium and potassium in the serum can give rise to electrolyte-related disorders such as hyponatremia and hyperkalemia, inflicting severe damage on the circulatory and nervous systems and potentially culminating in coma or even death [18]. Disruptions in the homeostasis of calcium and magnesium may either be the cause or the consequence of various pathological dysfunctions. Hypercalcemia can lead to abnormal renal calcium filtration, resulting in the formation of kidney stones. Hypomagnesemia, caused by a decline in serum magnesium levels, may trigger clinical manifestations such as nausea, weakness, and heart disease and further affect the central nervous system, leading to epilepsy or even coma [148].

### 3.3. The Applications of Metal Detection in Clinical Diagnosis

Given the established link between metal imbalance and human disease, the detection of specific metals has become an important diagnostic tool. Several common clinical applications of metal detection will be reviewed and discussed in this section.

Lead poisoning is a common occupational and prevalent disease caused by environmental pollution, which can lead to a series of complications such as impaired thyroid function and abdominal pain in patients [149,150]. It can be accurately diagnosed by measuring blood/urine lead levels [151]. If these levels exceed set thresholds, lead poisoning is confirmed.

Wilson disease, a copper metabolism disorder with hepatic and neural copper accumulation, is diagnosed via low serum ceruloplasmin, reduced urinary copper excretion, and increased hepatic copper. Otherwise, bladder cancer patients exhibit notably higher serum copper levels than healthy individuals, with significant correlations to VEGF and HIF-1 expression; thus, serum copper assays aid bladder cancer diagnosis [152,153].

Serum zinc levels are significantly reduced in patients with acute leukemia, potentially due to increased zinc uptake by tumor cells and enhanced enzyme activity [154]. Measuring serum zinc concentration can thus provide a reference for diagnosing this disease. Moreover, zinc deficiency is associated with several other cancers, such as esophageal squamous cell carcinoma, prostate cancer, and ovarian cancer, where patients also demonstrate significantly lower serum zinc levels than normal controls [155,156].

## 4. Absorption Mechanisms of Metal Elements

**Figure 3:**
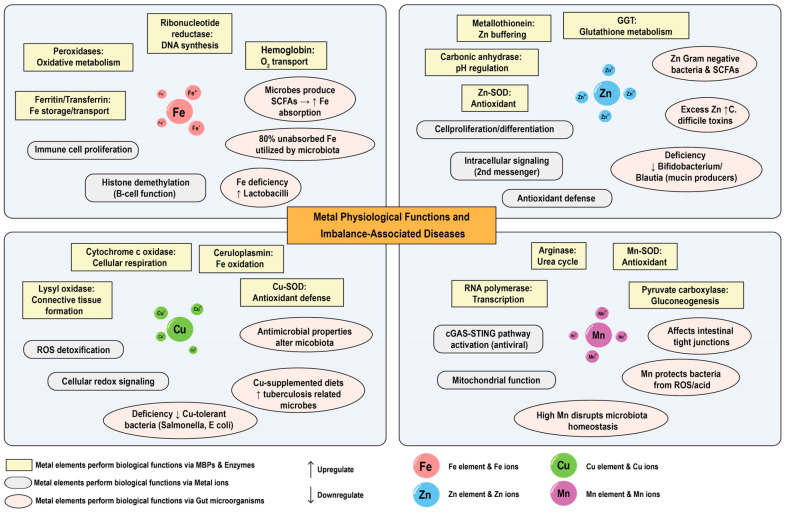
Physiological functionality of essential trace elements. Trace elements (Fe, Cu, Mn, and Zn) execute physiological functions via MBPs/Enzymes, metal ions, and gut microbiota regulation in mammalian systems.

Metal elements are predominantly acquired from dietary sources. These metal elements in food exist in the form of organic compounds (protein–organic complexes and enzyme cofactors) or inorganic salts. Metal elements are liberated into the gastrointestinal tract through the digestive process, chiefly absorbed by the proximal small intestine, and subsequently transported to all parts of the body via the portal vein and into the circulatory system. The absorption of metal elements is of crucial importance for maintaining the appropriate levels of metal elements in the body. Perturbation in the metal element content frequently leads to a spectrum of diseases associated with metal element deficiency or overload. Hence, mammals have evolved intricate mechanisms for the absorption, transport, and recycling of metal elements to regulate the homeostatic balance of metal element content within the body. This section focuses on elucidating the molecular basis of metal element absorption mechanisms and homeostatic maintenance in mammalian systems, particularly highlighting essential trace elements (Fe, Cu, Zn, and Mn), with Figure 3 synthesizing multi-element transport and metabolic pathways within organisms.

### 4.1. Absorption Mechanism of Iron Elements

Iron is a vital trace element that partakes in numerous essential physiological processes in mammals, such as oxygen transport, energy metabolism, and neurotransmitter synthesis. Iron in food predominantly exists in two forms: heme iron and non-heme (inorganic) iron. Non-heme iron assumes multiple forms, including soluble iron, iron in low-molecular-weight complexes, stored iron in ferritin, and iron in the catalytic centers of various other proteins, and it represents the principal form of iron in plant-derived foods [157]. Heme iron is the iron bound to porphyrin in hemoglobin and myoglobin. The iron atom is firmly bound within the porphyrin ring and is less prone to binding with other constituents in food, such as phytate, tannin, and polyphenols, and is more readily absorbed by the body in contrast to non-heme iron.

The majority of iron absorption transpires in the upper intestine, specifically the duodenum and proximal jejunum [158]. Duodenal epithelial cells express iron transport proteins that are involved in iron absorption. The body’s iron level is predominantly determined by the rate of iron absorption [159]. Ferrous iron transporter divalent metal ion transporter 1 (DMT1) is the primary non-heme iron transport protein. Mice with inactivated DMT1 genes in the intestine succumbed to severe anemia shortly after birth, attesting to the essential role of DMT1 in the absorption of non-heme iron in the intestine [160]. Most of the non-heme iron in food exists in the form of Fe (III) and must undergo reduction prior to being transported by DMT1. Duodenal cytochrome B (Dcytb) in the apical membrane of intestinal epithelial cells functions as an iron reductase, capable of reducing Fe (III) to Fe (II), which can then be specifically transported by DMT1 [161]. The iron export protein ferroportin 1 (FPN1) is expressed on the basolateral cell surface of intestinal cells and is responsible for transporting the Fe (II) absorbed and stored by intestinal cells into the plasma, where it binds to plasma transferrin (TF) and enters the circulatory system for distribution throughout the body [162].

Research on the absorption mechanism of heme iron is relatively scarce. It is hypothesized that heme iron may bind to the brush border of intestinal epithelial cells and enter the cells via endocytosis. Subsequently, the iron is released within the intestinal epithelial cells under the influence of heme oxygenases and is then transported to the plasma via FPN1 [157].

### 4.2. Absorption Mechanism of Copper Elements

Copper is essential for the proper functioning of numerous enzymes in biological systems and plays a critical role in regulating various cellular process [163]. Copper exists mainly in the form of Cu (II) and Cu (I) in mammals. Cu (II) in food is converted into Cu (I) by reductases on the surface of small intestinal cells and is then absorbed into the cells through copper transporter 1 (CTR1) [125]. Mice with the CTR1 gene knocked out in the intestine exhibited severe copper deficiency and early lethality, corroborating that CTR1 is the principal transport protein for copper absorption [164]. Although DMT1 also possesses the capacity to transport and absorb Cu (II) and Mg (II), the absorption of copper and manganese is not contingent on DMT1 [165]. In small intestinal cells, copper is released into the plasma through Cu transporting ATPase A (ATP7A). Most of it is then reintroduced into the liver via CTR1. In the liver, copper is distributed in three main ways: (1) a portion enters endogenous Cu-dependent proteins; (2) most copper ions bind to α2-globulin and are transported to organs and tissues to form ceruloplasmin; and (3) excess copper is transported to bile, with the majority being excreted through feces and a small fraction being reabsorbed in the digestive tract [125,165]. Bile excretion serves to eliminate excessive copper from the body. When the copper level in the peripheral circulation diminishes, ATP7A can release the copper stored in the liver into the bloodstream. A series of complex mechanisms collaborate to maintain the homeostatic balance of copper levels [166,167].

### 4.3. Absorption Mechanism of Manganese Elements

Manganese is a trace element essential for multiple physiological processes, such as bone formation, immune response, and carbohydrate metabolism and participates in metabolic reactions as a cofactor for various enzymes, including galactosyltransferase, Mn-superoxide dismutase, xanthine oxidase, and arginase [168]. Manganese is abundantly present in plant-derived foods, with grains, nuts, and vegetables serving as significant sources, while it is scarcely found in animal-derived foods. Similar to other metal elements, manganese is predominantly absorbed in the small intestine and possesses redundant absorption mechanisms. DMT1 can transport Mn (II), which is also a divalent metal ion, although this process is not obligatory [118]. Manganese ion transport proteins ZIP8 (SLC39A8) and ZIP14 (SLC39A14) present in small intestinal epithelial cells can mediate the transport of Zn (II) from the digestive tract into the cells [169]. Mutations in the gene encoding ZIP8 can severely disrupt Mn homeostasis and lead to severe Zn deficiency [170]. Mutations in the ZIP14 gene resulting in hypermanganemia, brain Mn accumulation, and juvenile Parkinson’s syndrome, also highlight the importance of ZIP14 in manganese absorption [171]. The knockdown of ZIP8, ZIP14, or DMT1 by siRNA transfection significantly reduced the reabsorption of Mn (II) by renal tubules, indicating that all three transport proteins contribute to the absorption of Mn(II) [172]. Approximately 20% of Mn (II) is oxidized to Mn (III) under the effect of the oxidase protein ceruloplasmin (CP) [173], Mn (III) binds to TF and is transported into the cells through endocytosis mediated by the transferrin receptor system (TfR). This Tf-dependent pathway is regarded as the primary mechanism for transporting Mn across the blood–brain barrier into the brain [174]. Most of the manganese in cells enter mitochondria through the mitochondrial uniporter complex (MCU) and serve as an enzyme cofactor. A portion of it is transported to the Golgi apparatus by secretory pathway Ca^2+^ -ATPase pump type 1 (SPCA1) and transmembrane protein (TMEM165). Excess Mn ions are transported from intestinal epithelial cells to the plasma through SLC30A10 and FPN1 [118].

### 4.4. Absorption Mechanism of Zinc Elements

Zinc is the second-most abundant trace element in the body, trailing only iron. The activities of over 300 enzymes and 1000 transcription factors are contingent upon zinc [175]. The homeostasis of zinc metabolism is regulated by an increase in absorption during zinc deficiency and excretion during zinc supplementation [176]. Zinc transport proteins are principally classified into two families: zinc/iron-regulated transporter-like proteins (ZIP) and zinc transporters (ZnTs). The former transports zinc from the extracellular environment and intracellular organelles (endoplasmic reticulum, mitochondria, and Golgi apparatus) to the cytoplasm, while the latter transports intracellular zinc to the extracellular space [177]. ZIP4 is the main carrier on the apical surface of intestinal epithelial cells and is a crucial protein for assimilating zinc from food. ZIP4 can regulate the intracellular zinc ion concentration through a feedback regulatory mechanism. When the extracellular zinc ion concentration is suboptimal, ZIP4 accumulates on the cell membrane surface to enhance zinc ion absorption. Conversely, when the intracellular zinc ion concentration rises, ZIP4 reduces its expression on the cell membrane through endocytosis and domain cleavage to curtail the influx of zinc ions into the cell [178]. ZIP5 and ZIP14, located on the basolateral side of intestinal epithelial cells, can transport endogenous zinc ions in the bloodstream into intestinal epithelial cells [179,180]. ZnT1, also situated on the basolateral membrane of intestinal epithelial cells, transports zinc ions from intestinal epithelial cells to the blood in the portal vein [181]. ZnT5B (SLC30A5B), positioned on the apical membrane of intestinal epithelial cells, exhibits bidirectional transport capabilities. It can both ferry zinc ions from the small intestinal lumen into intestinal epithelial cells and return intracellular zinc ions to the intestinal lumen. This function may serve as an additional regulatory mechanism for zinc homeostasis [130]. Zn (II), as a divalent metal ion, can also traverse the intestinal epithelial cell membrane through DMT1. However, irrespective of the transport protein employed, once zinc ions enter intestinal epithelial cells, ZnT1 will translocate the zinc ions from the epithelial cells to the blood in the portal vein [175]. Metallothioneins (MTs) constitute a family of proteins that play a pivotal role in the systemic regulation of trace elements [182]. In intestinal epithelial cells, cytoplasmic zinc ions bind to MTs, reducing the concentration of free zinc in the cells and releasing zinc ions when necessary to maintain intracellular zinc homeostasis [183]. MTs are also believed to mediate intracellular zinc transport and transfer to transport proteins [175].

### 4.5. Absorption Mechanisms of Other Macroelements

Calcium is the metal element present in the highest quantity of mammals, with the majority being stored in bones and teeth. Calcium ions partake in a diverse array of physiological processes, such as vascular dilation and contraction, muscle function, and cell signaling [184]. Calcium absorption occurs throughout the gastrointestinal tract, primarily via transcellular and paracellular transport. In the stomach, there is typically no calcium absorption, but the acidic environment furnished by gastric acid is conducive to the dissolution of calcium salts and subsequent intestinal absorption [185]. The small intestine is the principal site where most calcium ions are absorbed. In the transcellular calcium transport mechanism, calcium ions are initially transported into the cells by the transient receptor potential channel V6 (TRPV6) at the apical end of intestinal epithelial cells. Subsequently, calcium-binding proteins mediate the translocation of intracellular calcium to the basolateral side. Finally, multiple transport proteins on the basolateral membrane, such as the P-type primary active transport protein ATP2B1 and the secondary active transport protein SLC8A1, transport calcium ions out of small intestinal epithelial cells [186]. When the luminal calcium concentration far exceeds the plasma ionic calcium, the paracellular calcium transport mechanism predominates. Calcium ions passively diffuse along the electrochemical gradient through the paracellular space (lateral intercellular space) of intestinal epithelial cells [187], and the tight junctions between cells restrict the movement of ions in a charge- and size-selective manner [186].

Magnesium participates in over 600 enzyme-catalyzed reactions related to energy metabolism and protein synthesis and is the second most abundant cation in cells [188]. Magnesium absorption in the small intestine also principally relies on two pathways: the transcellular transport mechanism and the paracellular magnesium transport mechanism. Intestinal epithelial cells uptake magnesium ions on the intestinal mucosa through the transient receptor potential melastatin 6 homodimer channel (TRPM6) and TRPM7 [189]. Magnesium ions are actively transported and extruded out of intestinal epithelial cells through cystathionine β-synthase domain divalent metal cation transport mediator 4 (CNNM4) on the basolateral side. In the paracellular magnesium transport mechanism, the magnesium ions accomplish the absorption procedure by permeating through the magnesium ion channels that are modulated by claudin 7 (Cldn 7) and claudin 12 (Cldn 12) [190].

## 5. Dynamic Landscape of the Metal Elements in Mammals

The roles of metals in mammals and the absorption and transport mechanisms of metal elements have been elaborated in detail in the previous sections. However, the levels of metal elements within the mammalian organism are not static throughout their entire life cycle [22]. Different metal elements have varying contents in different life stages and provide different nutrients and functions for each stage to meet the diverse physiological requirements of mammals at various stages [191]. In general, the metals play multiple roles throughout the entire life cycle of mammals. Their contents and requirements change with the growth, development, and physiological state of the animals, ensuring that mammals can maintain normal physiological functions and a healthy state at each life stage.

### 5.1. Dynamics of Metal Elements in Milk During Lactation

Metal elements in milk are important factors affecting the early growth and development of infants [192]. Iron in breast milk is an important source of iron for newborn animals. Although its content is relatively low, its utilization rate is extremely high, providing an easily absorbable iron source for mammals in the early stage and ensuring life activities such as oxygen transport for infants during lactation [193,194]. Similarly, small amounts of trace metal elements like copper and zinc in milk also play a vital role in the development of the immune system of infants [194]. Moreover, calcium and magnesium, as the two metal elements with the highest contents in the milk of most mammals, are the key factors in assisting the formation of the skeleton and the development of the nervous system [194,195]. According to the stage of lactation, breast milk can be classified into three types: colostrum, transitional milk, and mature milk [196]. There are significant differences in the contents of metallic elements among these three types of milk. Studies found that the contents of iron, copper, manganese, zinc, calcium, and magnesium in the milk of different mammals change with the progress of lactation, showing diverse trends. These changes provide nutritional support for infants at different stages during the lactation.

In human milk, a research project that synthesized multiple breast milk studies from various countries indicated that the calcium content in breast milk decreases over lactation time, with the most rapid change occurring in the early lactation stage. Zinc has the highest content among all trace elements in milk. It starts at a relatively high level in the first week after childbirth and then drops rapidly in the first few months. Similar to zinc, the contents of copper and iron in breast milk also decline as lactation progresses [23,197]. Rats, being an important animal model for evaluating the nutrition of formula milk, show the same trends in their milk [23].

As an important economic animal and disease model, pigs exhibit significant differences in the changes in metal element contents in their milk compared to other animals. Moreover, large discrepancies are observed in the changes in metal elements in different pig-related studies [18,157]. However, it can be preliminarily agreed that the iron content in porcine colostrum is low and gradually increases during the early lactation process, which may be an important cause of iron-deficiency anemia in newborn piglets [198]. The calcium content in pig milk also rises with the progression of lactation, which is quite different from that of other animals [199]. The contents of the remaining metal elements generally show a downward trend during lactation.

Meanwhile, studies have found that in cow milk, except for the calcium content, which decreases with lactation progression, the differences in the contents of metal elements are not significant throughout the lactation period [200].

### 5.2. Dynamics of Metal Elements in Mammals with Age

In mammals, the changes in metal elements are closely related to age, and these changes exhibit unique dynamic characteristics in different organs [22,201].

With the increase in age, the levels and distributions of metal elements in organisms change significantly, reflecting the roles of metal elements in maintaining physiological functions [202]. Young individuals usually have poor metal absorption and excretion abilities, resulting in relatively high metal concentrations in their bodies, especially during the growth and development stage [203,204]. Studies have shown that the main elements of metals remain relatively stable throughout adult life, but with the increase in age, the levels of most elements (such as zinc, copper, selenium, manganese, magnesium, phosphorus, sulfur, and potassium) in tissues show a decreasing trend. In addition, some elements such as calcium increase with age in the kidneys and muscles, while iron shows a similar trend in the pancreas and testes [205]. Another study found that the main elements (such as electrolytes potassium, magnesium, and sodium) show significant uniformity and stability over time in different organs, but the trace elements (such as copper, iron, rubidium, and zinc) and ultratrace elements (such as selenium, cobalt, molybdenum, and cadmium) are more heterogeneously distributed in the body and change with age. Particularly in the brain, the change in metal concentration with age is the most significant, including the accumulation of iron and copper [22].

Meanwhile, there are differences in the requirements for metal elements among mammals at different age stages. During the juvenile stage, especially the lactation period, mammals have a relatively high demand for certain metal elements to support their rapid growth and development [206]. For instance, iron and zinc are crucial metal elements for supporting immune function and cell growth. The deficiency of these elements may lead to slowed growth and a decline in immunity [207]. Adult animals, on the other hand, have different requirements for metal elements in terms of maintaining physiological functions and metabolic balance. They may focus more on preventing the accumulation and toxic effects of heavy metals. Research has shown that when adult animals are exposed to metal pollution in the environment, the metal excretion mechanisms in their bodies become more active, which, to a certain extent, protects their health [208]. Therefore, understanding the changes and requirements of metal elements in mammals of different ages is conducive to formulating corresponding nutritional intervention and environmental protection strategies for promoting the health and growth of animals.

## 6. Conclusions and Perspectives

Mammalian metallomics research has provided a crucial perspective for deeply understanding the dynamic changes and biological functions of metal elements within the body. With the advancement of technology and continuous innovation in research methods, understanding of the roles of metal elements in physiological and pathological processes has become clearer. Metal elements are not only essential components of various enzymes but also participate in multiple biological processes such as cell signal transduction, antioxidant defense, and gene expression regulation. However, current metallomics research still faces various challenges and controversies. Results between different studies may vary due to differences in experimental design, sample selection, and analytical methods, which necessitates caution when interpreting the functions of metal elements. Therefore, it is particularly important to balance the viewpoints and findings of different studies. Future research should place greater emphasis on interdisciplinary cooperation, integrating knowledge from biology, chemistry, and medicine to comprehensively reveal the complex roles of metals in organisms.

In addition, multi-omics integration is becoming increasingly crucial in biomedical research, especially in understanding complex physiological mechanisms. By integrating data from metallomics and different omics levels (such as microbiomics, transcriptomics, and metabolomics), researchers can gain a more comprehensive understanding of the dynamic changes and interactions of metal elements within mammals, thus uncovering their pivotal roles in maintaining life activities. Meanwhile, numerous multi-omics data analysis methods have been proposed. These methods are based on deep learning and network architecture. For example, the multi-omics graph convolutional network (MOGONET) and hypergraph convolutional network (HyperTMO) have shown exceptional performance in classifying patients and identifying biomarkers.

Moving forward, the ongoing advancement of metallomics will not only enhance our understanding of the roles of metal elements in living organisms and their dynamic regulatory mechanisms but also provide new insights into the prevention and treatment of related diseases. Diverse metal detection and imaging technologies are expected to integrate with emerging fields such as machine learning, offering novel perspectives for clinical diagnostics. These developments will support early tumor detection and precision medicine while also providing valuable references for research in precise trace element nutrition. And it is important to acknowledge that our review has certain limitations. Our focus on specific aspects of mammalian metallomics means that we do not cover the entire breadth of the field. For example, we primarily concentrate on the detection of elemental concentrations and may not fully represent the significant contributions of bioinorganic chemistry and other metallomics techniques, such as the structural determination of biometal complexes through advanced spectroscopy and crystallography. Future research could benefit from a more integrated approach that combines our findings with these important areas of study.

## Figures and Tables

**Table 1:** Mammalian metallomics analytical techniques overview.

Category	Technique	Acronym	Detection Limit	Primary Applications	Advantages	Limitations
Spectroscopy	Flame Atomic Absorption Spectroscopy	FAAS	High ppb to ppm range	Milk, serum, feces, tissues	• Low cost • Simple operation • Wide application scope	• Single element only • Moderate sensitivity • Complex sample preparation
	Graphite Furnace AAS	GFAAS	Sub-ppb level (<1 ppb)	Trace metals in tissues/biofluids	• High sensitivity • Small sample volume	• Single element only • High cost • Slow analysis speed
	Inductively Coupled Plasma Optical Emission Spectrometry	ICP-OES	1–10 ppb (most elements)	Multi-element analysis (e.g., adrenal tissue, nutrition studies)	•Multi-element detection • Wide linear range • Robust performance	• Lower sensitivity vs. ICP-MS • Spectral interferences
	X-Ray Fluorescence	XRF	Percentage to sub-ppm levels	Non-destructive analysis (milk, liver, archaeological samples)	• Non-destructive • Minimal sample prep • Multi-element	• Poor trace sensitivity • Matrix effects • Bulk analysis only
Mass Spectrometry	Inductively Coupled Plasma Mass Spectrometry	ICP-MS	ppt level (parts-per-trillion)	Trace metals/isotopes in biomedicine (ASD biomarkers, cancer drug distribution)	• Highest sensitivity • Isotopic analysis • Ultra-wide linear range	• High cost • Complex sample digestion • Polyatomic interferences
Imaging	Laser Ablation ICP-MS	LA-ICP-MS	Sub-ppm to ppt (element-dependent)	Spatial metal distribution (e.g., Pb in brain, U/Th accumulation)	• 2D/3D elemental mapping • µm-scale spatial resolution • Semi-quantitative	• Semi-destructive • Requires matrix-matched standards • Limited depth profiling

**Table 2:** Associations between metal imbalances and diseases.

Metal Ion	Primary Absorption Site	Key Transporters	Absorption Mechanism	Regulation and Homeostasis	Associated Diseases
Iron	Duodenum, proximal jejunum	- DMT1 (apical uptake) - Dcytb (Fe^3+^ reductase) - FPN1 (basolateral export) - Transferrin (TF) (plasma transport)	- Non-heme Fe: Dietary Fe^3+^ reduced to Fe^2+^ by Dcytb→ DMT1-mediated uptake → intracellular storage → FPN1 export → binds TF in plasma. - Heme Fe: Endocytosed → degraded by heme oxygenase → Fe^2+^ exported via FPN1.	- Hepcidin regulates FPN1 degradation to control systemic Fe. - Excess Fe stored as ferritin.	Anemia (deficiency), neurodegeneration (excess), organ damage
Copper	Small intestine	- CTR1 (apical uptake) - ATP7A (basolateral export) - Ceruloplasmin (CP) (plasma transport)	Dietary Cu^2+^ reduced to Cu^+^ → CTR1 uptake → ATP7A export → binds CP or albumin in plasma.	- Liver redistributes Cu via ATP7B: • Bound to CP for circulation • Biliary excretion for excess Cu elimination.	Menkes disease (ATP7A defect), Wilson’s disease (ATP7B defect)
Manganese	Small intestine	- DMT1, ZIP8, ZIP14 (apical uptake) - SLC30A10, FPN1 (basolateral export) - Transferrin (TF) (Mn^3+^ transport)	Mn^2+^ uptake via DMT1/ZIP8/ZIP14 → oxidized to Mn^3+^ by ceruloplasmin → binds TF for systemic transport.	SLC30A10/FPN1 export excess Mn. - Accumulates in brain via TfR-mediated endocytosis.	Hypermanganemia, Parkinsonism-like syndromes
Zinc	Small intestine	- ZIP4 (apical uptake) - ZnT1(basolateral export) - ZIP5/ZIP14 (basolateral uptake) - Metallothionein (MT) (intracellular buffer)	ZIP4 mediates dietary Zn^2+^ uptake → Zn^2+^ bound to MT → ZnT1 exports Zn^2+^ to plasma. ZIP5/14 import Zn^2+^ from blood.	- MT sequesters excess Zn. - ZIP4 endocytosis downregulates absorption during Zn sufficiency.	Slowed growth, immune dysfunction, dermatitis
Calcium	Small intestine	- TRPV6 (apical uptake) - Calbindin (intracellular shuttle) - ATP2B1/SLC8A1 (basolateral export)	Transcellular: TRPV6 uptake → calbindin transport → ATP2B1/SLC8A1 export. Paracellular: Passive diffusion via tight junctions.	Vitamin D upregulates TRPV6/calbindin. PTH regulates renal/bone Ca^2+^ recycling.	Rickets, osteoporosis, hypercalcemia
Magnesium	Small intestine	- TRPM6/TRPM7 (apical uptake) - CNNM4 (basolateral export) - Claudin-7/12 (paracellular transport)	Transcellular: TRPM6/7 uptake → CNNM4 export. Paracellular: Claudin channels facilitate diffusion.	Kidney reabsorption via TRPM6/CNNMs maintains balance.	Hypomagnesemia, cardiac arrhythmias

## Data Availability

No new data were created or analyzed in this study. Data sharing is not applicable to this article.
